# Think Beyond the Obvious: Atypical Pulmonary Embolism Presentation in a Parkinson’s Disease Patient

**DOI:** 10.7759/cureus.88520

**Published:** 2025-07-22

**Authors:** Abdul Rehman, Syeda Zoya Chishti, Fazeelah Bibi

**Affiliations:** 1 Emergency Medicine Department, Pakistan Air Force Hospital, Islamabad, PAK; 2 Obstetrics and Gynecology Department, Pakistan Institute of Medical Sciences Hospital, Islamabad, PAK; 3 Emergency Department, Pakistan Institute of Medical Sciences Hospital, Islamabad, PAK

**Keywords:** atypical symptoms of pulmonary embolism, elderly fall, parkinson disease, pulmonary embolism, recurrent falls

## Abstract

Pulmonary embolism (PE), a potentially life-threatening condition, results from obstruction of the pulmonary arteries by thromboemboli. It typically presents with cough, dyspnea, pleuritic-type chest pain, hemoptysis, or hemodynamic instability. However, in patients with Parkinson’s disease (PD) or elderly patients, it may present with recurrent falls. Falls as a primary presenting symptom of PE are rare and underrecognized but should be considered in patients with PD, especially if the falls are new-onset, unexplained, or associated with signs of hemodynamic compromise. We report a case of an elderly patient with PD whose recent unexplained falls led to the diagnosis of PE, emphasizing the need for heightened clinical suspicion in this patient population, especially when presenting with unexplained falls in the emergency department. Missed diagnosis of PE can potentially lead to serious long-term morbidity and mortality.

## Introduction

Falls are a common and potentially serious complication of Parkinson’s disease (PD) due to gait disturbances, postural instability, motor fluctuations, or polypharmacy [1]. While most falls are attributed to neurodegeneration, acute deteriorations should always prompt evaluation for underlying secondary causes, including infections, metabolic derangements, or pulmonary or cardiovascular events. Pulmonary embolism (PE) may manifest atypically with nonspecific symptoms, such as recurrent unexplained falls, altered mental status, or generalized weakness. This atypical presentation of PE in patients with PD can pose a diagnostic challenge and can be mistakenly attributed to Parkinsonian features or medication side effects [2]. PE remains an underrecognized but critical differential in PD patients presenting with sudden-onset increase in falls, particularly in the absence of typical chest pain and classic respiratory symptoms. Emerging evidence suggests a heightened risk of venous thromboembolism (VTE), including PE, among individuals with PD, likely due to reduced mobility, autonomic dysfunction, and prothrombotic changes. Autopsy-based studies have identified PE as a significant and often underestimated cause of death in this population, while recent meta-analyses have reported a notably increased incidence of VTE in PD patients compared to the general population [3]. Timely diagnosis and treatment are important to prevent life-threatening complications such as right ventricular failure and sudden cardiac arrest [2]. 

## Case presentation

An 84-year-old man with idiopathic PD (noncompliant on levodopa therapy) visited our emergency department with head injury secondary to one of his three unexplained falls over the last three days. In addition, he was complaining of feeling lightheaded and dizzy all the time. His family reported that while he had occasional balance issues, this sudden increase in falls was very unusual for him. Furthermore, he mentioned mild fatigue but denied any chest pain, hemoptysis, shortness of breath, or cough. 

Assessment of his vital signs showed a heart rate of 68 bpm, normal blood pressure without any postural variations, and oxygen saturation of 94% on room air. Neurological exam revealed rigidity and bradykinesia (left-sided predominance), impaired postural reflexes, and an asymmetric resting tremor. Cardiopulmonary examination was normal without any signs of pedal edema; however, decreased air entry at the left lung base with mild dullness to percussion was noted. No calf tenderness or swelling was noticed on physical exam. 

On laboratory investigations, his complete blood count (CBC) revealed normocytic anemia and mild leukocytosis without neutrophilia. To evaluate the cause of his anemia, serum iron studies along with B12 and folate levels were sent; results of which reported low iron levels (Table 1).

**Table 1:** Laboratory investigations WBC: white blood cell; MCV: mean corpuscular volume; TF: transferrin

Test	Result	Normal range
WBC count	11.6 × 10^9 ^/L	4-10 × 10^9 ^/L
Hemoglobin	12.0 g/dl	13-17 g/dl
Platelets	350,000 × 10^9 ^/L	150-450 × 10^9^/L
MCV	87.4 fl	83-101 fl
Troponin I	0.12 ug/L	<0.04 ug/L
D-dimers	7650 ng/ml	0-800 ng/ml
Iron	9.5 umol/L	11-28 umol/L
Transferrin	1.9 g/L	2-3.6 g/L
Ferritin	381 ug/L	30-400 ug/L
TF saturation	20%	20-50%

**Figure 1:**
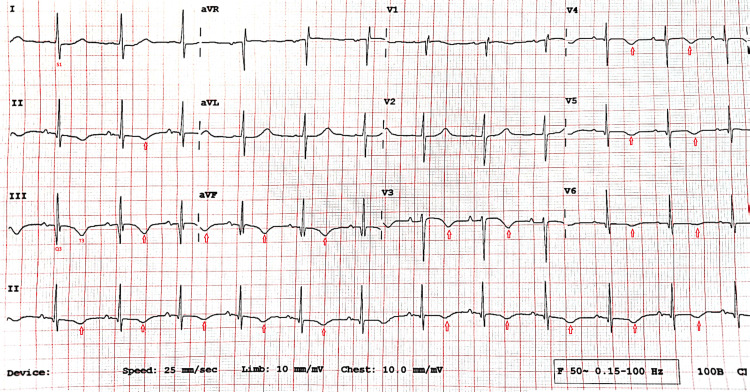
ECG showing S1Q3T3 pattern in lead l and lead lll along with simultaneous T wave inversion (red arrows) in precordial and inferior leads ECG: electrocardiography

Chest X-ray was remarkable for small left-sided pleural effusion with blunting of left costophrenic angle. To rule out any cardiac pathology as a potential cause of recurrent falls, electrocardiography (ECG) was performed, which revealed sinus rhythm with S1Q3T3 pattern and simultaneous T wave inversion in inferior and precordial leads (Figure 1). These changes were new compared to his previous ECG. Considering the new changes in ECG, D-dimer levels were obtained, which were reported elevated. No acute intracranial hemorrhage or features of stroke were reported on his CT brain.

**Figure 2:**
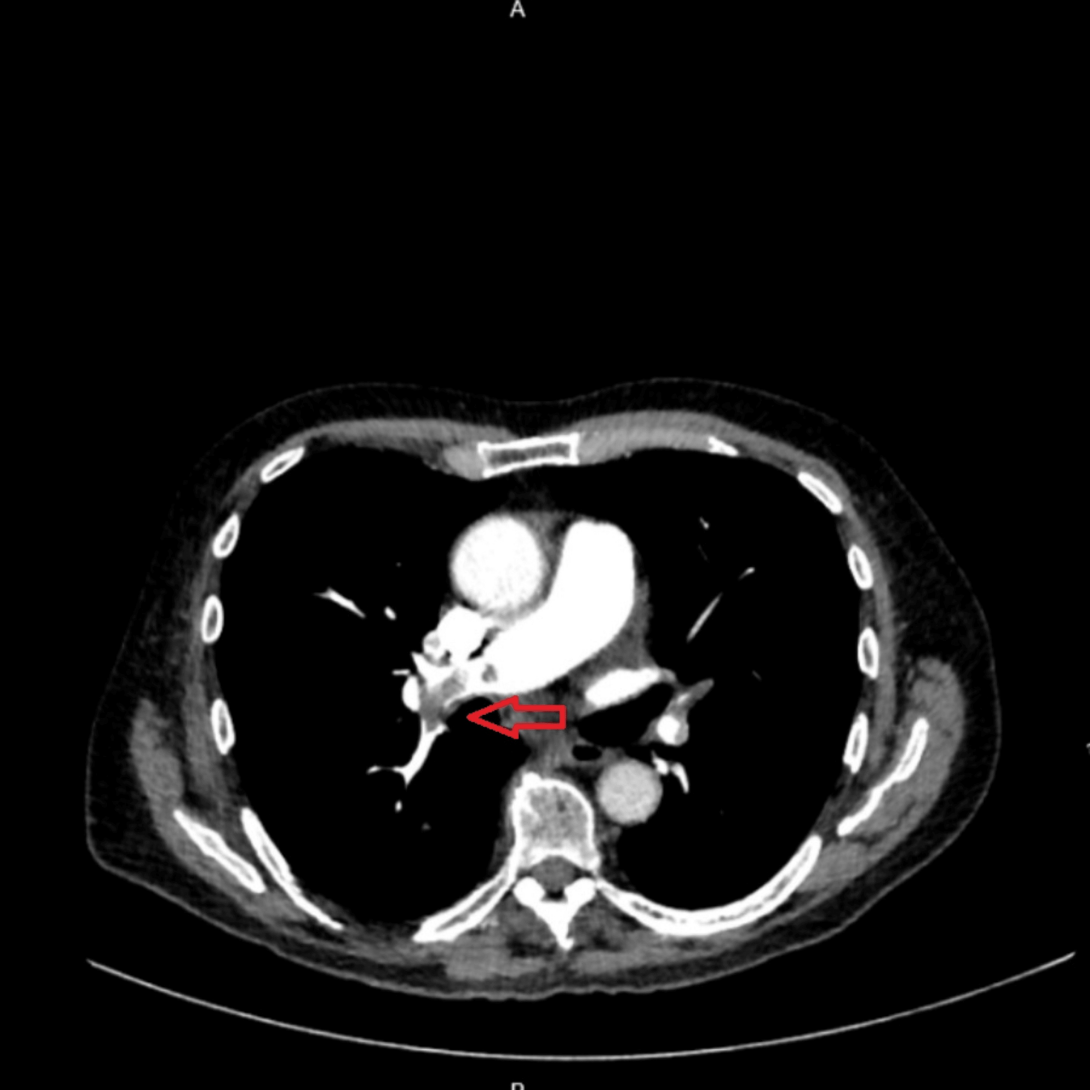
CT pulmonary angiogram showing embolism in the right pulmonary artery

**Figure 3:**
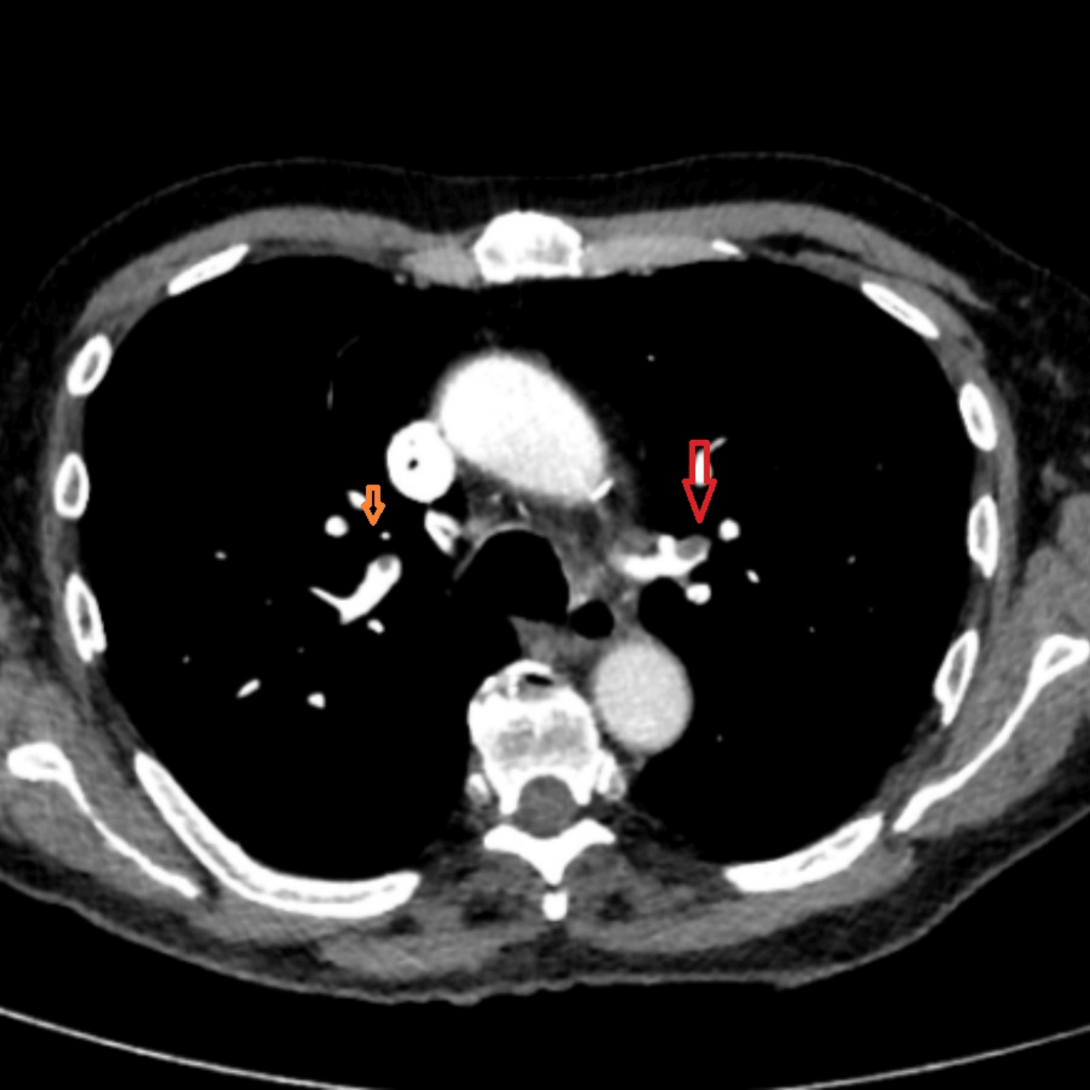
CT pulmonary angiogram showing pulmonary emboli in the right and left pulmonary arteries

Given the abnormal new ECG and chest X-ray findings, history of poorly controlled PD and medication noncompliance along with elevated D-dimer, a CT pulmonary angiogram was performed, which revealed extensive bilateral pulmonary emboli in left and right main pulmonary arteries distally along with small basal pericardial effusion and trace left sided pleural effusion (Figures 2, 3). Main pulmonary outflow trunk measured 31.8 mm, and reflux of contrast into intrahepatic inferior vena cava was seen. No evidence of saddle embolus was found. In addition, increased RV/LV ratio was present. Immediate anticoagulation with subcutaneous tinzaparin 175 IU/kg was commenced, and the patient was referred to the general medicine team for admission. In addition, mobility assessment by the physical therapy team and geriatric team was sought.

Our patient subsequently underwent CT thorax, abdomen, and pelvis with contrast, while admitted under medical team, to evaluate for the cause of his PE. His CT thorax revealed cardiomegaly with trace pericardial effusion, enlarged prostate, diverticulosis of sigmoid colon and descending colon, abnormal mural thickening of caecum, and a 10 mm lymph node near right common iliac artery. Subsequently, upper and lower GI endoscopies were performed, and biopsy samples were sent for analysis. Biopsy samples of cecum were reported positive for cecal adenocarcinoma, and he was referred to the general surgery team, where he underwent successful right hemicolectomy and made an uneventful recovery. He was then transferred to an acute rehabilitation facility and sent home with his original medication along with the addition of apixaban. At follow-up appointment after six weeks, the patient’s light-headedness and gait stability improved remarkably, and he reported no new falls after treatment.

## Discussion

The differential diagnosis of unexplained falls in the elderly population is broad [4]. Our case highlights that PE may present insidiously and atypically in patients with PD, particularly in the presence of undiagnosed underlying malignancy such as cecal adenocarcinoma. In this context, recurrent falls may represent the primary clinical manifestation. A limited number of atypical cases have been reported in the literature wherein PE presented with acute neuropsychiatric symptoms, such as delirium or acute psychosis, in individuals with PD [5,6]. PE should be considered in PD patients with abrupt increases in fall frequency, especially if accompanied by typical ECG findings of S1Q3T3 [7]. D-dimers, though nonspecific in older adults, can be a useful screening tool where clinical suspicion is high.

A high index of suspicion is important, as exemplified by our case where PE was not considered in the initial differentials due to symptoms being atypical, but turned out to be the culprit for the symptoms [8]. Apart from underlying asymptomatic cecal malignancy found on colonoscopy, the patient’s only risk factor that would have predisposed him to a PE would be extended periods of akinesia because of his medication noncompliance [9].

Falls are a prevalent and well-documented complication in patients with PD, often attributed to disease-related motor symptoms such as postural instability, bradykinesia, and rigidity [10]. However, clinicians must maintain a broad differential when evaluating new or worsening falls in PD, particularly when the presentation deviates from a patient’s usual disease trajectory. Our case highlights the importance of considering alternative, nonneurological etiologies, including cardiopulmonary causes, when assessing and evaluating falls in older adults with neurodegenerative disorders.

PE typically presents with symptoms such as dyspnea, chest pain, tachycardia, and hypoxia. However, atypical presentations are not uncommon, especially in the elderly and those with baseline neurological deficits [11]. In our case, the patient’s recurrent, unexplained falls were initially presumed to be due to progression of PD. The absence of classical cardiopulmonary symptoms can divert clinicians from considering PE in the differential diagnosis. This reflects a broader diagnostic challenge, where atypical presentations of PE may be misattributed to a patient’s underlying neurological condition or age-related frailty.

There are several plausible mechanisms by which PE may contribute to falls in this population. First, hypoxia and transient cerebral hypoperfusion resulting from PE can lead to syncope or near-syncope episodes resulting in falls. Second, PE, especially when massive, can lead to right heart strain or new-onset arrhythmias that may provoke hemodynamic instability, particularly in patients with autonomic dysfunction, common in PD. Additionally, reduced mobility and akinesia in PD patients are themselves risk factors for VTE due to prolonged immobility and venous stasis [11,12].

Our case underscores the necessity of considering PE in the differential diagnosis of recurrent, unexplained falls, especially in the absence of new focal neurological deficits or clear mechanical triggers. Early recognition and appropriate investigations (e.g., CT pulmonary angiography) are critical in preventing further morbidity and potentially fatal outcomes. Furthermore, it reinforces the importance of a thorough and multidisciplinary evaluation when encountering atypical presentations in a complex patient.

## Conclusions

This case illustrates the importance of thorough evaluation of unexplained recurrent falls in patients with PD. In patients with poorly controlled PD, recurrent falls may signal an underlying serious pathology such as undiagnosed cecal adenocarcinoma and PE, even in the absence of classic symptoms. Although falls are commonly linked to the progression of PD in this population, healthcare providers should always consider and maintain high suspicion for alternative diagnoses, as neurological manifestations of PD may mask the typical presentation, and relevant investigations should be obtained. In our case, PE presented without its typical respiratory or chest symptoms, making diagnosis particularly challenging. This emphasizes the value of a vast knowledge of typical ECG findings, e.g., presence of S1Q3T3 pattern and broad clinical perspective, especially in patients with PD and unexplained new ECG changes. Timely identification, investigation, and management of atypical presentations can significantly improve outcomes, even in those with chronic neurological disorders.
